# *AtPDS* overexpression in tomato: exposing unique patterns of carotenoid self‐regulation and an alternative strategy for the enhancement of fruit carotenoid content

**DOI:** 10.1111/pbi.12789

**Published:** 2017-09-11

**Authors:** Ryan P. McQuinn, Breanna Wong, James J. Giovannoni

**Affiliations:** ^1^ Department of Plant Biology Cornell University Ithaca NY USA; ^2^ Boyce Thompson Institute for Plant Research Cornell University Ithaca NY USA; ^3^ Robert W. Holley Center for Agriculture and Health USDA‐ARS Cornell University Ithaca NY USA; ^4^ Centre of Excellence in Plant Energy Biology Research School of Biology The Australian National University Canberra ACT Australia; ^5^ Present address: Australian Research Council Centre of Excellence in Plant Energy Biology Research School of Biology The Australian National University Canberra ACT Australia

**Keywords:** Phytoene desaturase, carotenoid isomerization, bioavailability, fruit ripening, tomato (*Solanum lycopersicum*)

## Abstract

The regulation of plant carotenogenesis is an active research area for both biological discovery and practical implementation. In tomato (*Solanum lycopersicum*), we demonstrate additional bottlenecks exist in the poly‐*cis‐*transformation of phytoene to lycopene in the context of ripening‐induced PSY1 expression and activity and reveal phytoene desaturase (PDS), as a target for manipulation towards elevated lycopene content in maturing tomato fruit. Overexpression of *Arabidopsis PDS*,* AtPDS,* elevated *PDS* transcript abundance in all aerial tissues resulting in both altered carotenoid accumulation and associated pathway gene expression in a tissue‐specific manner. Significant increases in downstream carotenoids (all‐*trans‐*lycopene and β‐carotene) and minimal changes in carotenogenic gene expression (carotenoid isomerase‐like 1, *CRTIL1*) suggest overexpression of heterologous *AtPDS* in tomato circumvents endogenous regulatory mechanism observed with previous strategies. In transgenic leaves, depletion of the PDS substrate, phytoene, was accompanied by minor, but significant increases in xanthophyll production. Alterations in the leaf carotenogenic transcript profile, including the upstream MEP pathway, were observed revealing unique feedback and feedforward regulatory mechanisms in response to *AtPDS* overexpression. *AtPDS* overexpression in the background of the *tangerine* (carotenoid isomerase, CRTISO) mutant exposes its potential in elevating downstream *cis*‐lycopene accumulation in ripe tomato fruit, as *cis*‐lycopene is more bioavailable yet less abundant than all‐*trans‐*lycopene in the wild‐type control. In summary, we demonstrate the limitation of PDS in ripening fruit, its utility in modifying carotenoid profiles towards improved quality, and reveal novel carotenoid pathway feedback regulation.

## Introduction

Carotenoids (e.g. lutein, β‐carotene and lycopene) and their catabolites (i.e. vitamin A and other apocarotenoids) are integral in the human diet as they ensure proper development and aid in the prevention of numerous age‐related diseases (Ip *et al*., 2013; Lian and Wang, 2008; Rhinn and Dollé, 2012; Sharoni *et al*., 2012). Humans’ inability to synthesize carotenoids *de novo* makes them dependent on plants as their primary source of dietary carotenoids, the acquisition of which is dependent upon both content in specific plant tissues and their ability to be absorbed in the digestive tract (bioavailability). The capacity of many fruits to accumulate elevated levels of carotenoids as they ripen makes fruits an important source of dietary carotenoids in many diets. Lycopene, the red carotenoid predominant in ripe tomatoes, has received increased attention as a target for manipulation due to evidence that lycopene‐derived apocarotenoid signals aid in cancer prevention (Ip *et al*., 2013; Lian and Wang, 2008).

**Figure 1:**
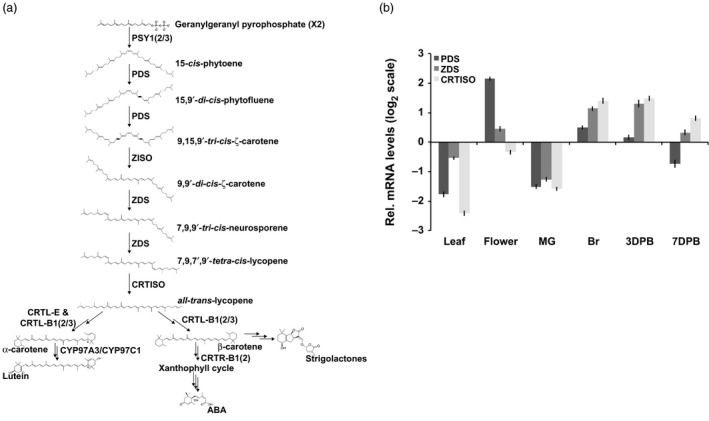
PDS is limiting in carotenogenesis during tomato fruit ripening. (a) Carotenoid biosynthetic pathway with enzymes abbreviated in bold: PSY1, 2 and 3; phytoene synthase 1, 2 and 3; PDS, phytoene desaturase; ZISO, ζ‐carotene isomerase; ZDS, ζ‐carotene desaturase; CRTISO, carotene isomerase; CRTL‐E (LCY‐E), lycopene ε‐cyclase; CRTL‐B1 and 2 (LCY‐B and BCYC, respectively), lycopene β‐cyclase; CYP97A3, carotene ε‐hydroxylase; CRTR‐B1 and 2, carotene β‐hydroxylase 1 and 2; ABA is abscisic acid. (b) Transcript levels of *PDS*,*ZDS* and *CRTISO* relative to a reference sample (combination of equal amounts of RNA from each tissue analysed) (*n *=* *3, in triplicate; error bars, ±SEM). Abbreviated tissues represent different stages of fruit ripening, mature green fruit, MG; breaker or onset of ripening, Br; and 3 and 7 days post breaker, 3DPB and 7DPB, respectively.

In plants, carotenoids are essential for light absorption in photosynthetic tissues and as provisional colorants in flowers and fruit facilitating the attraction of pollinators and seed dispersing organisms (reviewed in Nisar *et al*., 2015). The enhanced accumulation of carotenoids in ripening fruit is dependent on the switch from a photosynthetic to a nonphotosynthetic‐state, which embodies a remarkable transition of chloroplast to chromoplast‐rich cells, concurrent with strategic adjustments to the carotenoid profile within (Barry *et al*., 2008, 2012). Ripening‐associated carotenogenic gene expression in tomato is regulated by a complex symphony of transcription factors, the gaseous hormone ethylene and epigenome dynamics driving the flux of the pathway to all‐*trans‐*lycopene (and inhibition of its subsequent metabolism) conferring the typical red colour of tomato (Barry *et al*., 2005; Eriksson *et al*., 2004; Gallusci *et al*., 2016; Giovannoni *et al*., 2017; Martel *et al*., 2011; Vrebalov *et al*., 2002, 2009; Zhong *et al*., 2013). In the mature fruit, phytoene synthase (the first committed step in carotenogenesis) is encoded by the highly regulated *PSY1* gene and is the major limiting activity for carotenoid flux (Figure 1a) (Fraser *et al*., 2002). Once synthesized by PSY1, phytoene undergoes a poly‐*cis*‐transformation conferred by four desaturation reactions catalysed by phytoene desaturase (PDS) and ζ‐carotene desaturase (ZDS) and two isomerizations facilitated by ζ‐carotene isomerase (ZISO) and carotene isomerase (CRTISO) ultimately producing all‐*trans‐*lycopene (Figure 1a) (Alba *et al*., 2005; Fantini *et al*., 2013; Isaacson *et al*., 2002; Yu *et al*., 2011). Subsequent processing of all‐*trans‐*lycopene is prevented through downstream repression of lycopene ε‐ and β‐cyclases involved in synthesizing α‐carotene (ε,β‐rings), the precursor to lutein and the provitamin A, β‐carotene (β,β‐rings) (Figure 1a) (Ronen *et al*., 1999, 2000, respectively). These combined activities, tightly regulated to push carotenoid flux towards specific products in the ripe fruit, demonstrate finely tuned pathway regulation.

Multiple strategies have been pursued to enhance lycopene content in tomato deploying either plant or bacterial transgenes (reviewed in Fraser *et al*., 2009). In carotenoid deficient staple crops (e.g. rice and potato), the use of the bacterial carotenoid desaturase, CRTI, has proven effective due to its ability to catalyse all six reactions required to convert phytoene to all‐*trans*‐lycopene (Diretto *et al*., 2007; Misawa *et al*., 1990; Ye *et al*., 2000). Due to mechanisms yet to be defined, CRTI introduction *in planta* drives carotenogenesis beyond all‐*trans‐*lycopene to β‐carotene via induced lycopene β‐cyclase expression (Enfissi *et al*., 2017; Nogueira *et al*., 2013; Römer *et al*., 2000; Ye *et al*., 2000). In contrast to the successful applications in low carotenoid tissues, ectopic expression of *CRTI* was counterproductive for the enhancement of lycopene content in tomato fruit.

CRTI's structure has been solved facilitating characterization of its function as an FAD‐dependent oxidase/isomerase (Schaub *et al*., 2012). Two subsequent studies provided insight on the causal processes leading to β‐carotene enhancement in the tomato including CRTI protein localization (Nogueira *et al*., 2013) in the plastid and lycopene‐mediated pathway regulation (Enfissi *et al*., 2017). Remaining unclear is the potential for an uncontrolled elevation of a foreign carotenogenic protein (i.e. CRTI) to disrupt one or more of the many protein interactions involved in plant carotenogenesis, thus negatively affecting carotenoid gene expression (reviewed in Shumskaya and Wurtzel, 2013). Targeted manipulation of one or more of the four native plant enzymes carrying out limiting CRTI reactions in ripening fruit may facilitate enhanced all‐*trans*‐lycopene accumulation in an already high carotenoid tissue and provide insight into aspects of endogenous pathway regulation.

Here, we (i) identify PDS within the poly‐*cis‐*transformation of phytoene to lycopene as a useful target for manipulation towards carotenoid enhancement in ripening tomato fruit, (ii) fully characterize the effect of *PDS* overexpression on carotenoid content and carotenoid gene expression in aerial organs and (iii) demonstrate the utility of *PDS* overexpression combined with CRTISO repression via the *tangerine* (*t* ^3183^) mutation as a means to both elevate carotenoid levels and modify carotenoid accumulation profiles towards more bioavailable forms compared to wild‐type control tomatoes (Cooperstone *et al*., 2015).

## Results

### PDS becomes limiting once PSY1 is elevated during ripening

There is overwhelming evidence that PSY1 represents the major rate‐limiting step in ripening‐associated carotenogenesis (McQuinn *et al*., 2015; Nisar *et al*., 2015). The prevalence of intermediates in the poly‐*cis*‐transformation of 15‐*cis‐*phytoene to all‐*trans*‐lycopene as the fruit ripens suggests additional points of pathway limitation providing targets to develop alternate strategies for enhancing flux to desired downstream carotenoids. In terms of amino acid sequence identity and conserved domains, PDS and ZDS, enzymes are predicted to have evolved from CRTI, with 41% and 30% identity, respectively, and both contain the same functional domains, NAD‐binding domain and amine oxidase domain (NCBI Conserved Domains: http://www.ncbi.nlm.nih.gov/Structure/cdd/cdd.shtml) (Figure S1). In contrast, ZISO is considerably more distant and unrelated lacking both the NAD‐binding and amine oxidase domains and instead contains a nitric acid domain (NCBI Conserved Domains: http://www.ncbi.nlm.nih.gov/Structure/cdd/cdd.shtml) (Figure S1). Furthermore, analysis of protein structure (TOPCONS.net) indicates ZISO is the only enzyme in this part of the pathway predicted as a transmembrane protein with multiple transmembrane domains, consistent with previous reports (Beltrán *et al*., 2015). For these reasons and to gain a better understanding of the previously demonstrated metabolic constraints associated with CRTI overexpression, both PDS and ZDS were the focus of the efforts reported here, while ZISO, a more distant and distinct protein at the amino acid sequence and structural level (Beltrán *et al*., 2015), was left for later examination.

In an effort to identify the optimal target for increasing carotenoid pathway flux post‐PSY1 activity, expression patterns of *PDS* and *ZDS* were measured through four stages of fruit ripening (Mature Green, MG; breaker, Br; and 3 and 7 days postbreaker, 3DPB and 7DPB) using Quantitative Real‐Time PCR. *PDS* was the lowest expressed gene in the fruit as compared to *ZDS* and *CRTISO* (Figure 1b). While transcription does not determine enzyme activity, the elevated prevalence of PDS substrates (phytoene and phytofluene) compared to that of ZDS (9,9′di‐*cis‐*ζ‐carotene and neurosporene) in ripe fruit, as well as trace levels of PDS substrates observed in leaves (Table 1) suggest PDS activity, may be limiting during carotenogenesis. PDS shares the substrate, phytoene, with CRTI allowing for the additional assessment of potential feedback or feedforward effects from depletion of a specific substrate (i.e. phytoene) or over accumulation of a specific downstream product (i.e. all‐*trans‐*lycopene) as speculated in previous reports (Enfissi *et al*., 2017; Fantini *et al*., 2013; Kachanovsky *et al*., 2012). Together these observations suggested *PDS* as a high value target within the poly‐*cis‐*transformation for manipulation with the goal of elevating carotenoid accumulation.

**Table 1:** Available carotenoid content (μg/g FW) in the poly‐*cis*‐transformation of phytoene to all‐*trans*‐lycopene in leaves, flowers, and ripe fruit from wild type (AC) tomato plants. *(n *≥ 5)

	PDS		ZDS		CRTISO
	15′ *cis*‐phytoene	cis‐phytofluene	9,9′ di cis‐ζ‐carotene	*cis*‐neurosporene	Prolycopene
Leaf	0.07 ± 0.004	n.d.	n.d.	n.d.	n.d.
Flower	n.d.	n.d.	n.d.	n.d.	n.d.
Ripe fruit	1.72 ± 0.11	0.95 ± 0.06	0.23 ± 0.03	n.d.	n.d.

Carotenoid content is presented as μg/g fresh weight (FW). Values represent the mean of a minimum of five biological replicates ± standard error. n.d. denotes not detected.

### Heterologous overexpression of *Arabidopsis PDS* in tomato enhances downstream carotenoid content in ripe fruit

**Figure 2:**
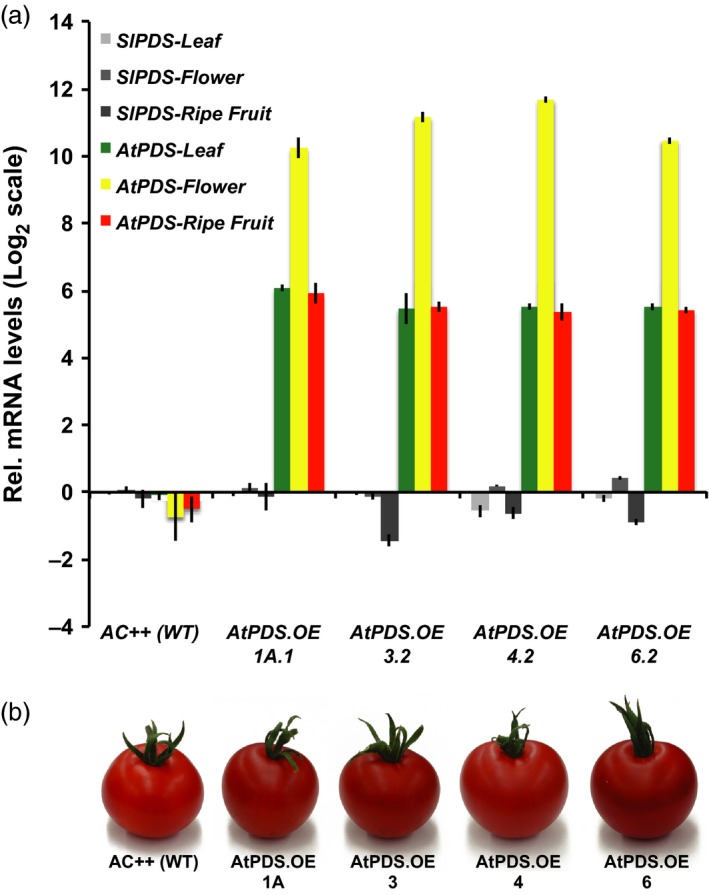
Overexpression of *AtPDS* enhances tomato fruit colour. (a) Transcript levels of tomato and *Arabidopsis PDS* (*SlPDS* and *AtPDS*, respectively) in stable, T_2_ generation *AtPDS* overexpression lines relative to the wild‐type control (cv. Ailsa Craig). Ripe fruit represents 7DPB (*n *=* *5, in triplicate; error bars, ±SEM). (b) Visual phenotypes of the chromoplast‐rich 7DPB fruit of the stable, T_2_ generation *AtPDS*.*OE* lines compared to the wild‐type control (cv. Ailsa Craig).

The potential for activating an endogenous targeted gene repression system (e.g. siRNA‐mediated gene silencing) in response to the introduction of a transgene can be exacerbated due to high nucleotide sequence similarity between the endogenous gene and the transgene. The coding sequence of *AtPDS* shares only 72% of sequence identity with that of the endogenous *SlPDS,* reducing the potential for activating DNA sequence homology‐based repression systems. For this reason, the *Arabidopsis* homolog of *PDS, AtPDS* (GenBank accession no. NM_202816) was deployed for overexpression rather than *SlPDS*, under the control of the constitutive CaMV 35S promoter in the wild‐type tomato background *cv*. Ailsa Craig (AC++). Four independent homozygous transgenic lines (i.e. *AtPDS1A.1*;* AtPDS3.2*;* AtPDS4.2*;* AtPDS6.2*) displaying elevated levels of *AtPDS* transcripts in the three aerial plant organs analysed (i.e. leaves, flowers and ripe fruits) were propagated to the T_2_ generation (Figure 2a). The observed minimal effect on endogenous *PDS* transcript levels (Figure 2a) indicated that any operating suppression systems had been bypassed. Ripe fruit from all four *AtPDS* overexpressing lines presented noticeably deeper red pigmentation compared to their wild‐type counterparts (Figure 2b).

**Figure 3:**
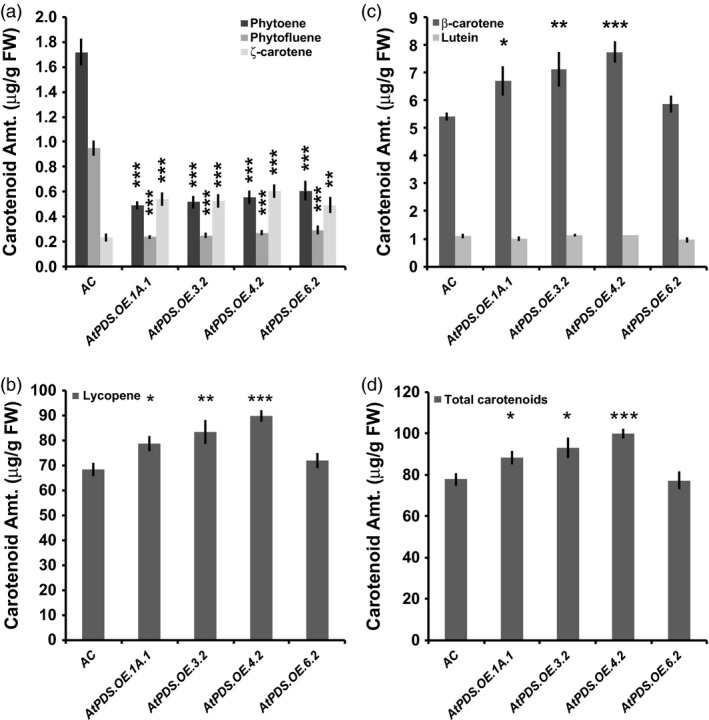
*AtPDS* overexpression pushes residual phytoene towards synthesis of downstream carotenoids in ripe tomato fruit. (a) Phytoene; phytofluene; and ζ‐carotene content (μg/g FW) in ripe fruit of stable, T_2_ generation *AtPDS*.*OE* lines compared to wild type (*n *=* *5, error bars ±SEM) (*P < 0.05; **P < 0.01; ***P < 0.001). (b) Lycopene content (μg/g FW) in ripe fruit of stable, T_2_ generation *AtPDS*.*OE* lines compared to wild type (*n *=* *5, error bars ±SEM) (*P < 0.05; **P < 0.01; ***P < 0.001). (c) β‐Carotene content (μg/g FW) in ripe fruit of stable, T_2_ generation *AtPDS*.*OE* lines compared to wild type (*n *=* *5, error bars ±SEM) (*P < 0.05; **P < 0.01; ***P < 0.001). (d) Total carotenoid content (μg/g FW) in ripe fruit of stable, T_2_ generation *AtPDS*.*OE* lines compared to wild type (*n *=* *5, error bars ±SEM) (*P < 0.05; **P < 0.01; ***P < 0.001).

Ripe fruit displayed the greatest increase in carotenoids downstream of PDS. Flowers and leaves had reduced and elevated lutein levels, respectively (Table 2). The metabolic effectiveness of *AtPDS* overexpression was evident in the reduction of steady‐state levels of phytoene and phytofluene, the substrate and intermediate of the PDS catalysed reaction, respectively, in both ripe tomato fruit (Table 2 and Figure 3a) and leaves (Table 2 and Figure S3). Total lycopene and β‐carotene were increased up to 31.1% and 42.8%, respectively (Table 2 and Figure 3b,c). With regard to the elevated total lycopene content, 31.1% equals an increase of 21.3 μg/g fresh weight in this already high lycopene tissue (Figure 3b). The observed increases in total lycopene and β‐carotene content in *AtPDS* overexpressing ripe are not a consequence of reduced fruit size (Figure S4). The largest metabolic shifts were observed in the immediate products of the PDS enzymatic reaction where total ζ‐carotene levels were >160% higher than in wild‐type fruit (Table 2 and Figure 3a).

**Table 2:** Carotenoid composition in ripe fruit (7DPB), flower petals, and leaves of stable T2 generatio AtPDS.OE tomato plants compared to wild type (AC) tomato plants

Genotype	Carotenoid composition (% of control ±SEM, *n* > 5)							
	Phytoene	Phytofluene	ζ‐carotene	Lycopene	ζ‐carotene	Lutein	Xanthophylls	Total carotenoids/xanthophylls
Fruit (7DPB)								
Ailsa Craig (WT)	100.0 ± 6.4	100.0 ± 6.5	100.0 ± 13.9	100.0 ± 3.9	100.0 ± 2.6	100.0 ± 7.2		100.0 ± 3.7
AtPDS.OE.1A.1	28.5 ± 1.9	24.9 ± 1.7	234.6 ± 23.8	110.1 ± 6.4	123.4 ± 9.7	90.9 ± 6.4		108.3 ± 6.3
AtPDS.OE.3.2	30.0 ± 3.0	26.1 ± 2.2	228.6 ± 24.1	121.9 ± 7.0	131.2 ± 11.5	102.9 ± 3.2		119.4 ± 6.3
AtPDS.OE.4.2	32.1 ± 3.2	28.5 ± 2.4	262.6 ± 24.3	131.1 ± 3.5	142.8 ± 6.9	101.6 ± 0.9		128.5 ± 3.0
AtPDS.OE.6.2	35.3 ± 4.7	30.7 ± 3.8	213.2 ± 28.3	100.8 ± 5.8	108.4 ± 5.7	86.9 ± 7.8		99.2 ± 5.5
Flower petals								
Ailsa Craig (WT)					100.0 ± 6.2	100.0 ± 3.5	100.0 ± 4.9	100.0 ± 4.5
AtPDS.OE.1A.1					110.9 ± 5.8	92.4 ± 3.3	106.3 ± 4.6	105.7 ± 4.2
AtPDS.OE.3.2					100.7 ± 6.5	92.3 ± 5.4	101.8 ± 4.0	101.2 ± 3.5
AtPDS.OE.4.2					95.5 ± 5.1	89.1 ± 3.1	103.8 ± 5.1	102.2 ± 4.7
AtPDS.OE.6.2					109.6 ± 4.2	95.4 ± 3.2	108.5 ± 2.6	107.8 ± 2.4
Leaves								
Ailsa Craig (WT)	Trace				100.0 ± 6.4	100.0 ± 2.9	100.0 ± 3.0	100.0 ± 3.3
AtPDS.OE.1A.1	n.d.				102.3 ± 6.4	118.2 ± 6.6	115.0 ± 4.5	113.3 ± 4.7
AtPDS.OE.3.2	n.d.				101.8 ± 4.6	110.9 ± 4.2	103.6 ± 3.5	104.0 ± 3.6
AtPDS.OE.4.2	n.d.				108.3 ± 2.8	117.6 ± 2.8	104.1 ± 2.7	106.6 ± 2.5
AtPDS.OE.6.2	n.d.				102.4 ± 4.1	106.4 ± 5.9	92.4 ± 5.0	96.1 ± 4.9

Carotenoid contents are presented as percent of control [*Ailsa Craig*(WT)] unless otherwise indicated. Values represent a mean of a minimum of 5 biological replicates ±SEM. Trace denotes detected at very low levels, n.d. denotes not detected. (Representative chromatograph and UV spectrums of carotenoids detected in the wildtype ripe tomato shown in Figure S2).

### *AtPDS* overexpression in ripening fruit resulted in altered transcription of *CRTIL1*

**Figure 4:**
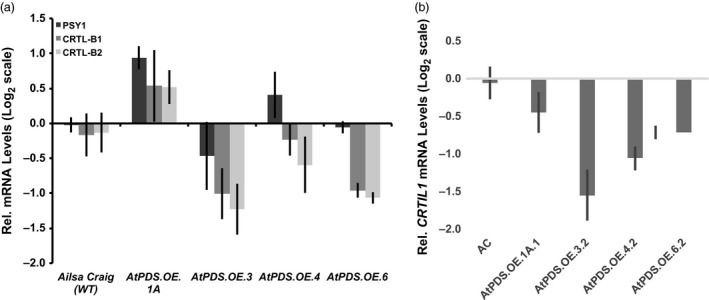
Carotenoid gene expression changes limited to *CRTIL1* in *AtPDS*.*OE* ripe fruit. (a) Transcript levels of *PSY1*,*CRTL‐B1* and *CRTL‐B2* in the ripe fruit of stable, T_2_ generation *AtPDS*.*OE* lines relative to the wild‐type control (cv. Ailsa Craig) (*n *=* *3, in triplicate; error bars ±SEM). (b) Transcript levels of *CRTIL1* in the ripe fruit of stable, T_2_ generation *AtPDS*.*OE* lines relative to the wild‐type control (cv. Ailsa Craig) (*n *=* *3, in triplicate; error bars ±SEM).

Considering the previously observed deregulation of transcripts associated with carotenoid biosynthesis in *CRTI* overexpressing fruit (Römer *et al*., 2000), it was necessary to quantify transcript abundance of genes throughout the biosynthetic pathway in ripe fruit of *AtPDS.OE* lines. A comprehensive analysis of the resulting carotenogenic transcript profile in *AtPDS.OE* ripe fruit was carried out via qRT–PCR with gene‐specific primers (Table S2) and compared to transcript abundance observed in the WT ripe fruit. Contrary to the observed off‐target changes in carotenogenic gene expression in *CRTI* overexpressing fruit first described in Römer *et al*. (2000), no consistent or statistically significant changes in *PSY1*,* CRTL‐B1* and *CRTL‐B2* gene expression were identified (Figure 4a). Analysis of relative transcript levels for the remaining carotenoid biosynthetic genes (*DXS; DXR; IDI1; PSY2, ZISO; ZDS; CRTISO; CRTIL1; CRTR‐B1; CRTR‐B2;* and *CRTL‐E*) further shows limited impact of elevated PDS on carotenoid pathway self‐regulation. A solitary significant change was observed in the reduction of *CRTIL1* transcript levels, a gene suggested to be involved in the isomerization of ζ‐carotenes (Fantini *et al*., 2013) (Figure 4b). These results indicate that PDS overexpression can provide a means for elevating carotenoid content in ripe fruit with limited feedback or feedforward effects on carotenoid gene expression and furthermore that *CRTIL1* responds to changes in flux through early steps in the pathway during tomato ripening.

### *AtPDS* overexpression in tomato leaves and flowers alters gene expression throughout the pathway

**Figure 5:**
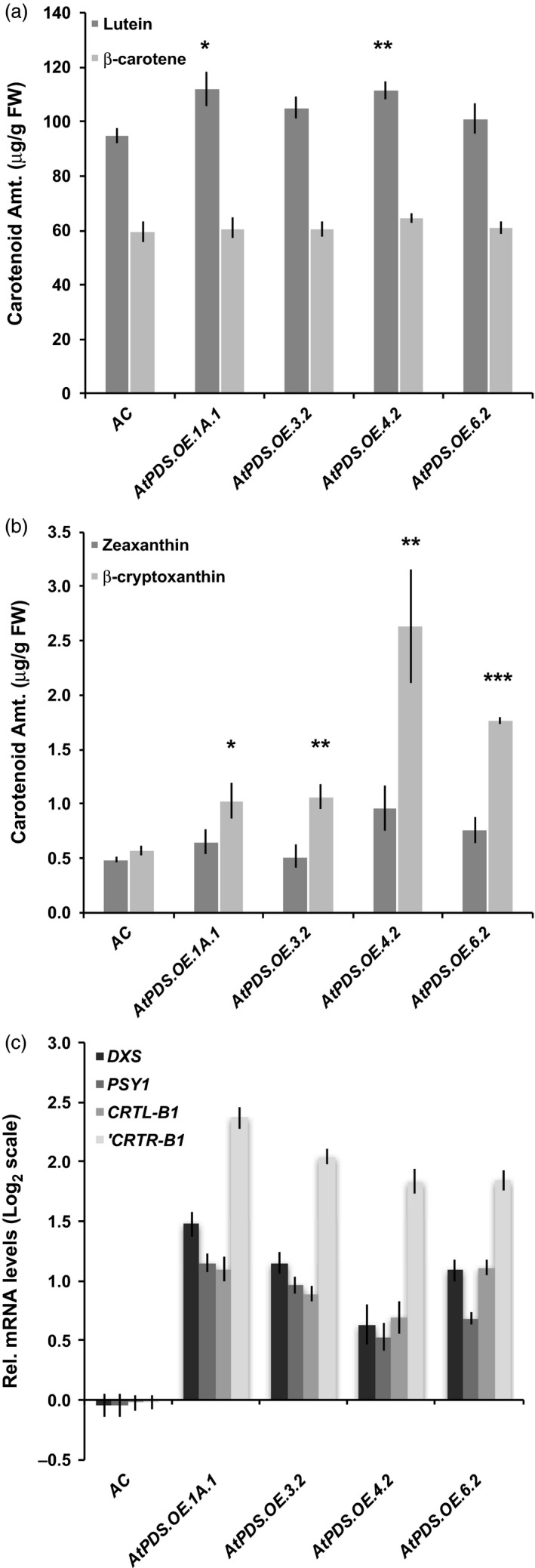
Enhanced photoprotective xanthophyll content in young leaves of *AtPDS*.*OE* lines. (a) Lutein and β‐carotene contents in young leaves of stable, T_2_ generation *AtPDS*.*OE* lines compared to wild type (*n *=* *5, error bars ±SEM) (*P < 0.05; **P < 0.01; ***P < 0.001). (b) Zeaxanthin and β‐cryptoxanthin contents in young leaves of stable, T_2_ generation *AtPDS*.*OE* lines compared to wild type (*n *=* *5, error bars ±SEM) (*P < 0.05; **P < 0.01; ***P < 0.001). (c) Endogenous regulation of carotenoid biosynthetic genes, deoxyxylulose 5‐phosphate (DXP) synthase, *DXS*; phytoene synthase 1, *PSY1*; chloroplast‐specific lycopene β‐cyclase *CRTL‐B1*; and chloroplast‐specific carotene β‐hydroxylase, *CRTR‐B1* (*n *=* *5, in triplicate; error bars ±SEM).

*AtPDS* overexpression in leaves enhanced carotenoid production in downstream xanthophylls, lutein, β‐cryptoxanthin and zeaxanthin to small but measurable degrees (Figure 5a,b). Analysis of transcript levels of carotenoid biosynthetic genes by qRT–PCR revealed that *AtPDS* overexpression in tomato leaves had broad regulatory consequences and influenced multiple steps throughout the carotenogenic pathway. The gene representing the rate‐limiting step of the MEP pathway (Rodríguez‐Concepción and Boronat, 2002), deoxyxylulose 5‐phosphate (DXP) synthase (*DXS*) was induced approximately twofold in the *AtPDS.OE* lines compared to the wild‐type control (Figure 5c). *PSY1* was also elevated in leaves, but to a lesser amount than *DXS* in *AtPDS.OE* leaves (Figure 5c). Lycopene β‐cyclase 1 (*CRTL‐B1*) and β‐carotene‐hydroxylase 1 (*CRTR‐B1*) were both expressed at higher levels in the *AtPDS.OE* leaves (Figure 5c). *CRTR‐B1* increased the most, (threefold to fourfold), and in combination with other changes in carotenoid gene expression provides insight into the mechanism underlying the elevated lutein, β‐cryptoxanthin and zeaxanthin content (Figure 5). β‐Carotene content remained unchanged (Figure 5).

**Figure 6:**
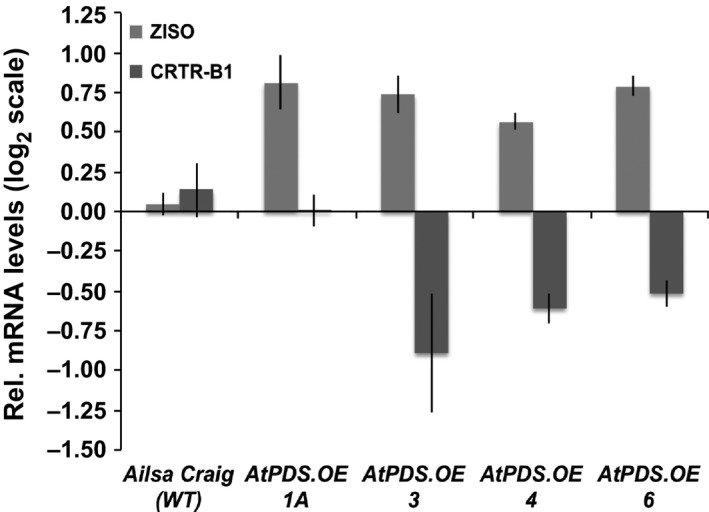
Deregulation of *ZISO* and *CRTR‐B1* in *AtPDS*.*OE* flowers. Transcript levels of *ZISO* and *CRTR‐B1* in the anthesis flowers of stable, T_2_ generation *AtPDS*.*OE* lines relative to the wild‐type control (cv. Ailsa Craig) (*n *=* *3, in triplicate; error bars ±SEM).

Carotenoid biosynthetic gene expression analysis of the chromoplast‐rich flowers displayed limited alterations in response to heterologous PDS expression, and this was again even more limited in the maturing fruit as noted above. The gene encoding the step immediately following *PDS*, ζ‐carotene isomerase (*ZISO*), was elevated approximately twofold compared to wild‐type flowers (Figure 6). *CRTR‐B1* was reduced slightly in the *AtPDS.OE* flowers, potentially limiting the ability to increase xanthophyll content of the petals and anthers as confirmed by HPLC (Figure 6).

### *AtPDS* overexpression enhances *cis*‐lycopene accumulation in *tangerine* (*CRTISO*) mutant ripe fruit

In an effort to further amplify the value of the metabolic shift towards *cis*‐carotenoids in *tangerine* (*t* ^3183^) mutant fruit, both *AtPDS3.2* and the *high pigment 1* (*hp1*) mutations were crossed independently into the *t* ^3183^ background. The *hp1* mutation in the tomato *DAMAGED DNA BINDING PROTEIN1* (*DDB1*) gene was selected as it confers an increase in plastid number resulting in elevated accumulation of carotenoids in ripe fruit (Liu *et al*., 2004), a means of carotenoid elevation distinct from the direct increase of pathway flux conferred by *AtPDS* overexpression in tomato. *hp1;t* ^3183^ double‐mutant lines were also developed as a standard for comparison of metabolic pathway shift.

**Figure 7:**
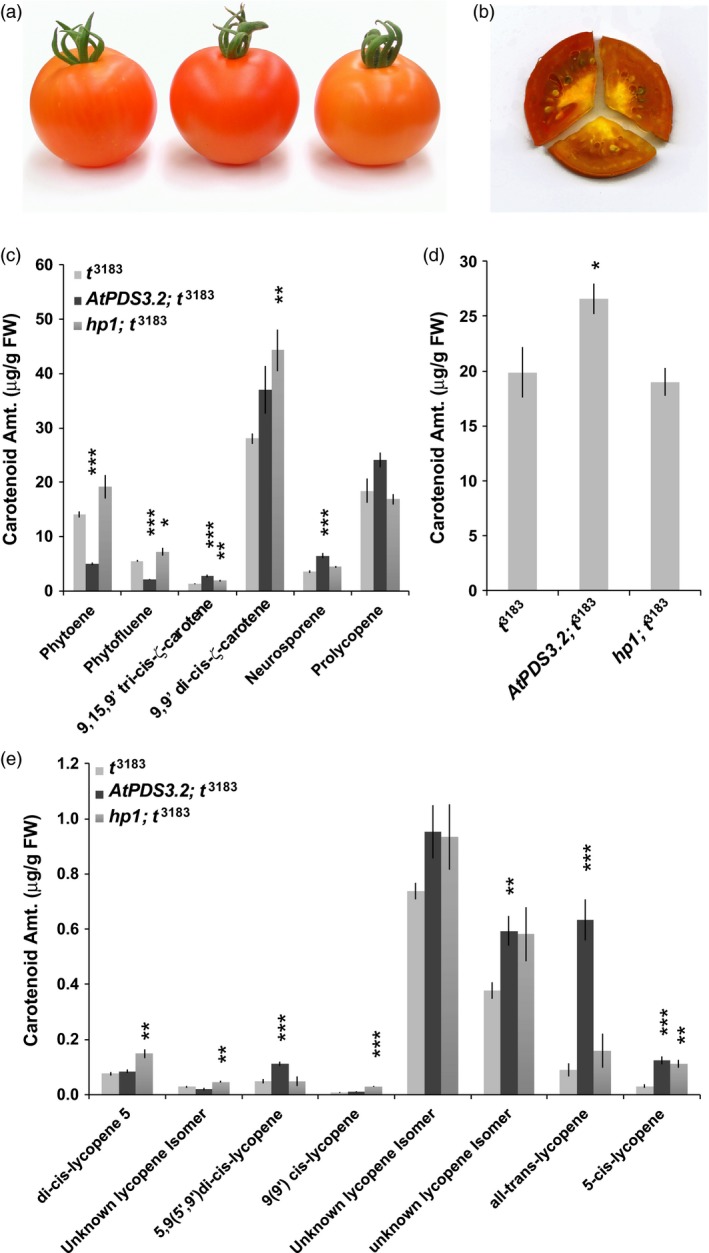
Overexpression of *AtPDS* enhances downstream *cis*‐carotenoid accumulation in the *tangerine* mutant. (a) External visual phenotype of *tangerine* (*t* ^3183^) compared to *AtPDS3.2*;* t* ^3183^ and *hp1*;* t* ^3183^ double mutants. (b) Cross sections of ripe fruit display internal pericarp pigmentation in *t* ^3183^, *AtPDS3.2*; *t* ^3183^ and *hp1*;* t* ^3183^. (c) *Cis‐*carotenoid content (μg/g FW) from the poly‐*cis*‐transformation of phytoene to all*‐trans‐*lycopene in *t* ^3183^, *AtPDS3.2*;* t* ^3183^ and *hp1*;* t* ^3183^ 7DPB fruit (*n *=* *5; error bars, ±SEM) (*P < 0.05; **P < 0.01; ***P < 0.001). (d) Total lycopene content in 7DPB fruit of *t* ^3183^, *AtPDS3.2*;* t* ^3183^ and *hp1*;* t* ^3183^ (*n *=* *5; error bars, ±SEM) (*P < 0.05; **P < 0.01; ***P < 0.001). (e) *Cis‐* and all‐*trans‐*lycopene content (μg/g FW) accumulating in 7DPB fruit of *t* ^3183^, *AtPDS3.2*;* t* ^3183^ and *hp1*;* t* ^3183^ (*n *=* *5; error bars, ±SEM) (*P < 0.05; **P < 0.01; ***P < 0.001). All carotenoid names identify peaks with similar characteristics and retention timing as those identified in Melendez‐Martinez et al. (2013).

Plants homozygous for the *tangerine (t* ^3183^ *)* mutation and positive for the *AtPDS* transgene or *hp1* were selected in F2 populations. All mutations and transgenes were nearly isogenic in the inbred Ailsa Craig background to minimize additional genetic variation in double‐mutant lines. Phenotypic and HPLC analyses were carried out on the ripe fruit of the *AtPDS3.2;t* ^3183^ double mutant, the *t* ^3183^ parent line and the *hp1*;*t* ^3183^ double mutant. Compared to both the parental *t* ^3183^ and *hp1*;*t* ^3183^ ripe fruit, *AtPDS3.2;t* ^3183^ fruit were darker in colour (Figure 7a). The enhanced pigmentation associated with *AtPDS3.2;t* ^3813^ was observed throughout the pericarp of cut fruit (Figure 7b).

HPLC analysis revealed that *AtPDS* overexpression reduced steady‐state levels of phytoene and phytofluene by 64.8% and 61.8%, respectively, consistent with reductions observed when overexpressed in the wild‐type background (Figure 7c and Table 2; Figure S5). AtPDS efficiency appears to be influenced by tissue type as *AtPDS3.2;t* ^3183^ anthesis flowers only displayed a 30% reduction relative to *t* ^3183^ phytoene and phytofluene levels (Figures 7c and S6). Both *AtPDS* overexpression and the *hp1* mutation led to a similar increase in 9,9′‐di‐*cis‐*ζ‐carotene content when combined with *t* ^3183^ (Figures 7c and S5), while *AtPDS3.2;t* ^3183^ alone displayed significant increases in products downstream of 9,9′‐di‐*cis*‐ζ‐carotene when compared to both *t* ^3183^ and *hp1*;*t* ^3183^ (Figures 7c and S5). Total neurosporene accumulates up to 84.5% higher in *AtPDS3.2;t* ^3183^ compared to *t* ^3183^ and 44.5% higher than *hp1*;*t* ^3183^ (Figures 7c and S5). Additionally, tetra‐*cis*‐lycopene (prolycopene) levels are 30.3% and 42.2% higher than in *t* ^3183^ and *hp1*;*t* ^3183^, respectively (Figures 7c and S5).

Total lycopene levels followed the same trend as prolycopene in the three genotypes with *AtPDS3.2;t* ^3183^ displaying the highest levels (Figures 7d and S5). Subsequent lycopene isomer identification and quantification in the ripe fruit demonstrates genotype‐specific effects on particular lycopene isomers (Figures 7e and S7; Table S2). Introduction of *AtPDS* overexpression and the *hp1* mutation in *t* ^3183^ conferred elevations in two unidentified lycopene isomers and 5‐*cis*‐lycopene relative to the *t* ^3183^ parental line (Figure 7e). *AtPDS3.2;t* ^3183^ tomatoes accumulate 2 and 7 times more 5,9(5′,9′) di‐*cis*‐lycopene and all‐*trans‐*lycopene, respectively, compared to levels found in *t* ^3183^ tomatoes, while levels remain relatively unchanged in *hp1*;*t* ^3183^ (Figure 7e). The presence of genotype‐specific changes in *cis*‐lycopene isomers and elevated total lycopene content observed in *AtPDS3.2;t* ^3183^ demonstrates the potential for targeting PDS in a means that bypasses endogenous regulation in concert with CRTISO to enhance bioavailable *cis‐*lycopene isomers in tomato.

## Discussion

### *AtPDS* overexpression effectively enhanced carotenoid accumulation in fruit and significantly alters the carotenoid gene expression pattern in a tissue‐specific manner

Carotenogenesis is heavily regulated regardless of plant organ, with PSY1 activity the major rate‐limiting step in ripening‐associated carotenoid biosynthesis (McQuinn *et al*., 2015; Nisar *et al*., 2015). This study demonstrates that in the context of normal ripening induction and function of PSY1, PDS activity becomes limiting and when elevated through deployment of a heterologous transgene, can significantly increase downstream carotenoid (i.e. ζ‐carotene, lycopene and β‐carotene) accumulation and total carotenoid output (Table 2 and Figure 3). Moreover, when overexpressed in the ripening tomato, PDS metabolized 70% of the remaining phytoene in the plastid ultimately contributing to the elevated downstream carotenoids including the desired all‐*trans‐*lycopene (Table 2). The largest relative increase in carotenoid content was in ζ‐carotene suggesting that as PDS activity was elevated, ZISO and ZDS now became limiting. These two genes thus present interesting targets for further carotenoid enhancement in ripening fruit with elevated PDS.

Importantly and in contrast to previous observations with transgenic tomatoes expressing the bacterial *CRTI* (Enfissi *et al*., 2017; Nogueira *et al*., 2013; Römer *et al*., 2000), total carotenoid content was significantly elevated in *AtPDS.OE* ripe fruit (Table 2 and Figure 3d). Furthermore, *PSY1* transcripts did not decrease, nor did lycopene β‐cyclase transcripts (*CRTL‐B1* and *CRTL‐B2*) increase across the four independent transgenic lines (Figure 4). The lack of feedback regulation within the carotenogenic pathway may rely on the fact that an enzyme native to plants, PDS, was introduced rather than a more distant bacterial enzyme, CRTI. Given that both PDS and CRTI considerably deplete the phytoene pool in the plastids (based on observed steady‐state levels), the observed feedback regulation does not appear directly attributable to a response to lower substrate availability. It has recently been suggested that this response may be due to an elevation in lycopene content (Enfissi *et al*., 2017). PDS increases the accumulation of all‐*trans‐*lycopene in ripe tomato plastids more than with CRTI overexpression (Table 2 and Figure 3b)( Enfissi *et al*., 2017; Nogueira *et al*., 2013; Römer *et al*., 2000). While the lack of elevated lycopene in response to CRTI is due to enhanced lycopene β‐cyclase activity, additional studies with *CRTB* (bacterial *PSY*) overexpressed in ripening tomatoes increased lycopene, absent increases in lycopene β‐cyclase (*CRTL‐B1* and *CRTL‐B2*) expression (Fraser *et al*., 2002; Nogueira *et al*., 2013). It is possible that misregulation throughout the pathway in response to CRTI may be through means other than or in addition to elevated lycopene.

While PDS evolved from CRTI, PDS and CRTI function in contrast to each other with regard to maintenance of the FAD redox state and may differentially influence carotenoid pathway regulation in the ripening tomato fruit. It has been established that the plastid terminal oxidase, PTOX and the plastoquinone (PQ) pool controls the redox state and function of PDS in plants (Carol *et al*., 1999; Joët *et al*., 2002; Josse *et al*., 2003; Norris *et al*., 1995; Yu *et al*., 2014). Moreover, while in photosynthetic chloroplasts, alternative mechanisms appear to aid in the maintenance of PDS's redox state, in nonphotosynthetic chromoplasts, PDS is solely dependent on PTOX and the PQ pool (Shahbazi *et al*., 2007). Norflurazon inhibits PDS activity by maintaining the FAD within PDS in a reduced state once it is lodged in the active site (Gemmecker *et al*., 2015). In contrast, CRTI cannot be inhibited by norflurazon (Sandmann and Fraser, 1993), and as suggested by Schaub *et al*. (2012), it remains unclear whether CRTI can be oxidized similarly to PDS by PQ *in planta*. Therefore, in contrast to PDS overexpression, ripe fruit expressed CRTI protein may have the potential to disrupt the redox state of the PQ pool providing an alternative explanation for the observed carotenoid pathway misregulation.

*AtPDS* overexpression successfully circumvents the negative feedback mechanisms observed in response to bacterial CRTI. Indeed, *AtPDS* overexpression associated elevation of lycopene and β‐carotene in the T_2_ generation ripe fruit is improved compared to values observed with other strategies (Enfissi *et al*., 2004; Fraser *et al*., 2007). Lycopene content resulting from *AtPDS* overexpression is higher than reported in DXS overexpression lines in the T_1_ generation, while total carotenoid content stays comparable (Enfissi *et al*., 2004). Observations by Fraser *et al*. (2007) demonstrated that PSY1 overexpression increases phytoene content in ripe transgenic fruit, but has no overall effect of the steady‐state levels of lycopene when compared to wild‐type fruit. In contrast, *AtPDS* overexpression overcomes the apparent limitation in the pathway at the PDS step and increases the steady‐state levels of lycopene in the ripe transgenic tomato fruit.

**Figure 8:**
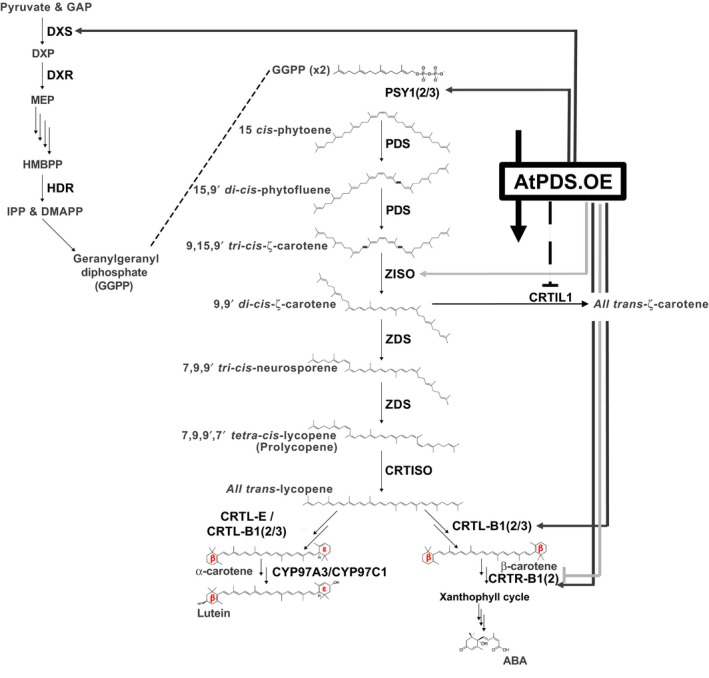
Model of feedback and feedforward regulation induced by AtPDS overexpression in ripe fruit, leaves and flowers. Enzymes within the simplified MEP pathway are abbreviated in bold. DXS, deoxyxylulose 5‐phosphate (DXP) synthase; DXR, DXP reductase; HDR, 4‐hydroxy‐3‐methylbut‐2‐enyl diphosphate reductase. Enzyme products are abbreviated as follows: GAP, glyceraldehyde 3‐phosphate; DXP, deoxyxylulose 5‐phosphate; MEP, methyl‐d‐erythritol 4‐phosphate; HMBPP, 4‐hydroxy‐3‐methylbut‐2‐enyl diphosphate; IPP, isopentenyl diphosphate; DMAPP, dimethylallyl diphosphate; and GGPP, geranylgeranyl diphosphate. Carotenoid biosynthetic enzymes are abbreviated in bold: PSY1, 2 and 3, phytoene synthase 1, 2 and 3; PDS, phytoene desaturase; ZISO, ζ‐carotene isomerase; ZDS, ζ‐carotene desaturase; CRTISO, carotene isomerase; CRTIL1, CRTISO‐like 1; CRTL‐E (LCY‐E), lycopene ε‐cyclase; CRTL‐B1 and 2 (LCY‐B and BCYC, respectively), lycopene β‐cyclase; CYP97A3, carotene ε‐hydroxylase; CRTR‐B1 and 2, carotene β‐hydroxylase 1 and 2; and ABA is abscisic acid. Black dotted bar represents negative impacts on gene expression in response to *AtPDS* overexpression in ripe fruit. Dark grey arrows represent positive impacts by AtPDS overexpression in leaves. Light grey arrows/bars represent positive/negative impacts by AtPDS overexpression in anthesis flowers.

Many examples of pathway self‐regulation have been reported including, but not limited to, the feedback regulation observed when CRTI is overexpressed in tomato fruit (Enfissi *et al*., 2017; Nogueira *et al*., 2013; Römer *et al*., 2000) and epistatic regulation of PSY1 in the spontaneous *tangerine* (*CRTISO*) mutation (Kachanovsky *et al*., 2012; and reviewed in Cazzonelli and Pogson, 2010). Therefore, it was not surprising to observe tissue‐specific transcriptional perturbations of carotenogenic genes in response to *AtPDS* overexpression (Figure 8). Interestingly, artificially increasing flux via elevated PDS triggered major alterations in gene expression throughout the carotenoid biosynthetic pathway in leaves (Figures 5 and 8), while modifications in transgenic ripe fruit across all four independent *AtPDS.OE* lines were limited to a small change in *CRTIL1* (Figures 4b and 8). Additionally, minor unique changes in pathway gene expression were also detected in the flowers but with no significant alterations in carotenoid content (Figures 6 and 8).

Considering the important protective roles of carotenoids in chloroplast stress tolerance, it is understandable that the carotenoid pathway would be more responsive to perturbations within leaf chloroplasts. The transcript induction resulting from *AtPDS* overexpression was similar, yet distinct from gene expression changes in tomato leaves expressing *CRTI* reported in Nogueira *et al*. (2013). In the AtPDS.OE leaves, induced *DXS*,* PSY1*,* CRTL‐B1* and *CRTR‐B1* transcripts and subsequent elevation of xanthophylls (i.e. lutein, β‐cryptoxanthin and zeaxanthin) are consistent with previous reports of elevated zeaxanthin levels in cyanobacteria from elevated PDS protein (Chamovitz *et al*., 1993). However, the minimal changes observed in xanthophyll accumulation in response to the elevation in gene expression throughout the pathway are intriguing. Similar observations have been observed when manipulating PSY in photosynthetic tissues of Arabidopsis (Lätari *et al*., 2015; Maass *et al*., 2009; Zhou *et al*., 2015). Whether via overexpression of PSY or the PSY activating ORANGE protein, carotenoid enrichment was limited to nonphotosynthetic tissues (Maass *et al*., 2009; Zhou *et al*., 2015). Lätari *et al*. (2015) demonstrate that overexpression of PSY in the *ccd4* mutant background results in xanthophyll accumulation to toxic levels, suggesting a role for CCD4 in controlling xanthophyll accumulation. PDS‐associated enrichment of xanthophylls in tomato leaves may be similarly restricted via CCD4.

### A strategy for enhancing lycopene content and bioavailability in tomato fruit

Bioavailability as it is most commonly defined (Macrae *et al*., 1993) equally depends on both the accessibility of the nutrient to the human body and its absorption properties therein. Impaired accessibility of carotenoids can negatively influence the overall effectiveness of carotenoid enhanced crops in addressing food security and nutritional concerns. Recent reports have suggested chromoplast ultrastructure and carotenoid sequestration effect the overall bioavailability of carotenoids, where bioavailability is improved via sequestration of carotenoids in a lipid‐dissolved liquid–crystalline state (Schweiggert *et al*., 2012, 2014). However, in tomatoes and other all‐*trans‐*lycopene‐rich fruits, all‐*trans‐*lycopene forms long crystalline aggregates of approximately 15 μm in length in the chromoplasts inhibiting its accessibility (Cooperstone *et al*., 2015; Schweiggert *et al*., 2012, 2014).

Chromoplast ultrastructure has been observed to be dependent on carotenoid composition (Harris and Spurr, 1969). Furthermore, an abundance of *cis*‐carotenoids in chromoplast‐rich tissues has been suggested to promote lipid‐associated sequestration of carotenoids (Ben‐Amotz *et al*., 1988). Microscopic analysis of chromoplasts in *tangerine* mutant (*t* ^3183^) fruit demonstrates that the abundance of *cis*‐lycopene isomers promotes carotenoid sequestration in globular lipid‐rich structures improving the accessibility of all lycopene isomers (Ben‐Amotz *et al*., 1988; Cooperstone *et al*., 2015). Therefore, overexpression of *AtPDS* in the *tangerine* mutant (*t* ^3183^) of tomato provides a unique and effective strategy capable of elevating total lycopene content without complementing the *tangerine* mutation, thereby accumulating higher levels of the bioavailable *cis*‐lycopene isomers (e.g. tetra‐*cis*‐lycopene; 5,9‐di‐*cis*‐lycopene; 5‐*cis*‐lycopene, etc.) and maintaining enhanced accessibility of carotenoids, including all‐ *trans*‐lycopene, sequestered in a lipid‐dissolved liquid crystalline state (Ben‐Amotz *et al*., 1988; Cooperstone *et al*., 2015; Schweiggert *et al*., 2012, 2014). The strategy overexpressing *AtPDS* in the *t* ^3183^ background reported here provides an initial step towards the enhancement of bioavailable carotenoids in fruit crops enabled in part by regulatory insights revealed through heterologous expression of PDS in tomato fruit.

## Experimental procedures

### Plant materials and growth conditions

Wild‐type control, *tangerine* single mutant (LA3183) and *tangerine; high‐pigment1* double*‐*mutant (LA3367) seed (*Solanum lycopersicum* cv Ailsa Craig) were obtained from the Tomato Genetics Resource Center, UC Davis (http://tgrc.ucdavis.edu/). Plants were grown in glasshouses at the Guterman Bioclimate Laboratory and Greenhouse Complex, Cornell University, Ithaca, NY. All plants were grown under natural light conditions consisting of 16‐h day/8‐h nights. *AtPDS* overexpression transgenic lines were carried on to the T2 generation. T2 generation plants showing the strongest consistent phenotypes were used for crosses to make double mutants/transgenics with *tangerine*. Leaf tissue was harvested from the 4th, 5th and 6th leaves from the meristem of a two‐month‐old plant. Whole petals and anthers were harvested from flowers at the point of anthesis/pollination. Ripe fruits were collected 7 days after tagging fruit at the first sign of colour change at the blossom end (i.e. breaker stage). The tomato fruit ripening time course is represented by fruit collected at four stages of fruit ripening (Mature Green, MG; breaker or onset of ripening, Br; and 3 [light red] and 7 days [full red] postbreaker, 3DPB and 7DPB, respectively).

### Development of overexpression constructs and plant transformation

The *AtPDS* overexpression (AtPDS.OE) construct was generated as described previously in Gleave (1992) using the pART7 and pART27 binary vector strategy. RNA from *Arabidopsis thaliana* (accession Columbia‐0) leaf tissue was converted to cDNA via iScript™ cDNA synthesis kit (Cat. No. 170‐8891; Bio‐Rad; Hercules, CA). Resulting cDNA was used to amplify the full‐length AtPDS ORF via FastStart High‐fidelity PCR system (Cat No. 04‐738‐292‐001; Roche Applied Sciences; Indianapolis, IN) with AtPDS‐OE‐KpnI.for and AtPDS‐OE‐XbaI.rev primers (Table S1). The resulting AtPDS.OE construct was sequence‐verified and transformed into *S. lycopersicum* cv Ailsa Craig by *Agrobacterium tumafaciens* (strain LBA‐4404) as previously described (Van Eck *et al*., 2006).

### DNA isolation and zygosity and copy number analysis

Genomic DNA was isolated from fresh meristematic leaf tissue as previously described (Barry *et al*., 2005). Verification of insertion events in T0 plants was confirmed via PCR using primers specific for the 35S promoter in the pHELLSGATE 2 vector, 35S‐for and 35S‐rev (Table S1). Zygosity and copy number were determined in the T1 generation via Quantitative PCR relative to the single copy polygalacturonase 2a gene (PG2a, Accession No. X04583) using a modified protocol described in Haurogné *et al*. (2007) Quantitative PCR was performed in 5 μL reactions containing 2.5 μL SYBR^®^ Green PCR Master Mix (Cat No. 4309155; Applied Biosystems, Foster City, CA); 0.75 μL of 10 μm of each primer; and 1 μL of gDNA using an ABI PRISM™ 7900HT Sequence Detection System (Applied Biosystems, Foster City, CA) under the following reaction conditions: 48 °C for 2 min; 95 °C for 10 min; and 40 cycles of 95 °C for 15 s and 60 °C for 1 min. Amplification was followed by a dissociation curve to verify specificity of the 35S promoter‐specific and PG gDNA‐specific primers used (Table S1).

Identification of positive double mutants/transgenics was performed using a forward and reverse primers in the interior of the 35S promoter and AtPDS ORF (35S‐internal‐FOR and AtPDS‐internal‐REV, respectively) and primers spanning the deletion in the *tangerine* mutant (i.e. tangerine.mut‐for and tangerine.mut‐rev) (Table S1). All positive double mutants/transgenics were further verified by gene expression via qRT–PCR and identification of appropriate flower and fruit phenotypes (Isaacson *et al*., 2002).

### RNA isolation and quantitative RT–PCR analysis

Total RNA was isolated from all previously described tissue using a modified protocol from the RNeasy Minikit (Cat. No 74106, Qiagen Sciences, Germantown, MD). RNA was extracted from 200 to 300 mg of frozen powdered tissue via the addition of 800 μL modified RLT buffer (4 m guanidine isothiocyanate; 0.2 m sodium acetate, pH 5.2; 25 mm EDTA; 2.5% (w/v) PVP‐40) containing 1% (v/v) β‐mercaptoethanol and 2% sarcosine and separated upon the addition of 800 μL chloroform. Total RNA in the upper aqueous phase was collected on the Econospin™ minispin column (Cat. No. 1920250; Epoch Life Sciences, Missouri City, TX). The column was washed twice with 500 μL of RPE buffer (Cat. No. 1018013; Qiagen Sciences; MD), and RNA was eluted with 360 μL of nuclease‐free water. DNase treatment of total RNA was achieved by adding 40 μL DNase reaction buffer (60 mm MgCl2; 400 mm Tris, pH 7.5) and 3 μL of RQ1 DNase enzyme (Cat. No. M6101; Promega, Fitchburg, WI) and allowed to incubate for 45 min at 37 °C. Finally, RNA samples were washed with an equal volume of phenol: chloroform solution (500 g phenol crystals; 500 mL chloroform; 20 mL isoamyl alcohol; 0.5 g 8‐hydroxy‐quinoline; equilibrated with 100 mm Tris, pH 8.0) and lastly with an equal volume of chloroform. Total RNA was then precipitated overnight at −20 °C and resuspended in 50–100 μL of DEPC‐treated water.

Quantitative Real‐time PCR was performed using the Power SYBR^®^ Green RNA‐to‐C_T_™ 1‐Step Kit (Cat No. 4309169; Applied Biosystems, Foster City, CA) in a 5 μL reaction volume (2.5 μL 2X Master Mix; 1 μm forward and reverse primers; 1 μL of total RNA; 0.46 μL DEPC‐treated water; 0.04 μL RT enzyme mix). All tissue samples were represented by three biological replicates, each being carried out in triplicate. Gene‐specific primers were checked for efficiency using wild‐type RNA (for primer sequence see Table S1). To be able to apply the standard curve method described in Applied Biosystems User Bulletin # 2 (http://www3.appliedbiosystems.com/cms/groups/mcb_support/documents/generaldocuments/cms_040980.pdf), a standard curve was included on each plate for the specific gene being analysed using wild‐type RNA (serial dilutions: 50 ng; 5 ng; 0.5 ng; 0.05 ng) in triplicates. For each gene analysis, template‐free and negative‐RT controls were included. Real‐time PCRs were carried out using an ABI PRISM™ 7900HT Sequence Detection System (Applied Biosystems, CA) under the following reaction conditions: reverse transcription at 48 °C for 30 min; enzyme activation at 95 °C for 10 min; followed by 40 cycles of 95 °C for 15 s and 60 °C for 1 min. A dissociation curve was added at the end of the run for verification of primer specificity.

ABI PRISM™ SDS version 2.3 software (Applied Biosystems, CA) was used to determine gene‐specific threshold cycles (C_T_) using the endogenous reference (18S rRNA) for every sample. C_T_s were extracted, and the standard curve method (Applied Biosystems, 1997) was applied to calculate relative mRNA levels in comparison with the wild‐type control or the reference sample (equal volume and concentration of RNA from each tissue combined), which was employed for the wild‐type differential expression profile.

### Carotenoid extraction and analysis

Carotenoids were extracted from 200 mg of frozen tomato pericarp using a modified protocol from Alba *et al*. (2005). Carotenoid content of anthesis flowers was extracted from frozen petals and anthers of a single flower. The frozen tissue was homogenized in a Savant FP120 Fast Prep machine first with 15 mg Mg‐carbonate and 450 μL of tetrahydrofuran (THF) twice and then a third time with 450 μL of methanol containing 2,6‐Di‐tert‐butyl‐4‐methylphenol (MeOH/BHT). The homogenate was then transferred and filtered through Spin‐X centrifuge filters (0.45‐mm nylon filter; Corning/Costar #8170; Corning Inc., Corning, NY). Tissue debris was re‐extracted with an additional 500 μL of THF to ensure complete extraction of carotenoids. The carotenoid/nonpolar phase was separated from the aqueous phase through two separation steps, first with 375 μL of petroleum ether and 150 μL of 25% NaCl and next with 500 μL of petroleum ether. The two upper phase aliquots were combined and dried down in a vacufuge (Eppendorf). Prior to drying the flower extracts, xanthophylls went through a saponification as described in Galpaz *et al*. (2006). The dried extract from fruit was resuspended in 250 μL of ethyl acetate, while dried flower extracts were resuspended in 500 μL. All solvents used were HPLC grade.

Carotenoid analysis was carried out using a Dionex HPLC machine (ThermoFisher, Waltham, MA) (P680 HPLC pump, ASI‐100 automated sample injector, PDA‐100 photodiode array detector) and Chromeleon (v6.40) software package (ThermoFisher, Waltham, MA). Separation of carotenoids was achieved under a polar to nonpolar gradient (0–5 min 100% methanol: 0.1% ammonium acetate; 6‐ to 25‐min ramp to 4% methanol: ammonium acetate and 96% methyl t‐butyl ether; 26‐ to 30‐min ramp to 100% methanol: ammonium acetate; 31–35 min 100% methanol: ammonium acetate) through a guard cartridge (YMC carotenoid S‐5, 4.0 × 20 mm DC guard; Waters, Milford, MA), C_30_ column (YMC carotenoid S‐5, 4.6 × 250 mm; Waters, Milford, MA) assembly. Seven channels were used for data acquisition, channel 1 (286 nm); channel 2 (348 nm); channel 3 (398 nm); channel 4 (428 nm); channel 5 (437 nm) and channel 6 (450 nm); channel 7 (464 nm). Peak identification was performed as described in Alba *et al*. (2005). Lycopene isomers identification was determined relevant to results in Melendez‐Martinez *et al*. (2013) (Figure S6 and Table S2).

## Supporting information


**Figure S1** Assessment of protein structure of tomato PDS; ZDS; and ZISO compared to CRTI from bacteria.
**Figure S2** Representative chromatograph (285 nm—best wavelength to show all peaks) of carotenoids detected in the wild type ripe tomato.
**Figure S3** Absence of phytoene (μg/g FW) accumulation in young leaves of *AtPDS.OE.1A, AtPDS.OE.3, AtPDS.OE.4,* and *AtPDS.OE.6* (*n* = 5, Error bars ±SEM).
**Figure S4** Fruit size in terms of mass (g) of *AtPDS.OE.1A, AtPDS.OE.3, AtPDS.OE.4,* and *AtPDS.OE.6* ripe fruit compared to wild type ripe fruit (*n* > 10, Error bars ±SEM).
**Figure S5** Chromatographs with carotenoids detected in the *tangerine* (*t* ^3183^); *AtPDS3.2;t* ^3183^; and *hp1*;*t* ^3183^ ripe tomatoes.
**Figure S6** Tissue type negatively affects the extent of *AtPDS* over‐expression in *tangerine* mutant flower.
**Figure S7** Representative chromatograph (454 nm) of stereoisomers of lycopene red, all‐*trans‐*lycopene standard (4 °C) and blue, stereomutated all‐*trans‐*lycopene (80 °C for 1 h).
**Table S1** Primer sequences
**Table S2** Identification of lycopene stereoisomers detected in the *tangerine* (*t* ^3183^) ripe fruit based on artificial stereomutation of All‐*trans‐*lycopene standard according to Melendez *et al*. (2013)
