# Supplementary material for: AtPDS overexpression in tomato: exposing unique patterns of carotenoid self‐regulation and an alternative strategy for the enhancement of fruit carotenoid content
Source: Plant Biotechnol J. 2017 Sep 11;16(2):482–94. doi: 10.1111/pbi.12789 (PMC5787846; doi:10.1111/pbi.12789)
Supplement: Supplementary file 1 — Figure S1 Assessment of protein structure of tomato PDS; ZDS; and ZISO compared to CRTI from bacteria. Figure S2 Representative chromatograph (285 nm—best wavelength to show all peaks) of carotenoids detected in the wild type ripe tomato. Figure S3 Absence of phytoene (μg/g FW) accumulation in young leaves of AtPDS.OE.1A, AtPDS.OE.3, AtPDS.OE.4, and AtPDS.OE.6 (n = 5, Error bars ±SEM). Figure S4 Fruit size in terms of mass (g) of AtPDS.OE.1A, AtPDS.OE.3, AtPDS.OE.4, and AtPDS.OE.6 ripe fruit compared to wild type ripe fruit (n > 10, Error bars ±SEM). Figure S5 Chromatographs with carotenoids detected in the tangerine (t 3183); AtPDS3.2;t 3183; and hp1;t 3183 ripe tomatoes. Figure S6 Tissue type negatively affects the extent of AtPDS over‐expression in tangerine mutant flower. Figure S7 Representative chromatograph (454 nm) of stereoisomers of lycopene red, all‐trans‐lycopene standard (4 °C) and blue, stereomutated all‐trans‐lycopene (80 °C for 1 h). Table S1 Primer sequences Table S2 Identification of lycopene stereoisomers detected in the tangerine (t 3183) ripe fruit based on artificial stereomutation of All‐trans‐lycopene standard according to Melendez et al. (2013) [file PBI-16-482-s001.docx]

Supplemental Data

| **Table S2.** Identification of lycopene stereoisomers detected in the *tangerine* (*t*^3183^) ripe fruit based on artificial stereomutation of All-*trans-*lycopene standard according to Melendez *et al.* (2013). | | | | | | |
| --- | --- | --- | --- | --- | --- | --- |
| **Lycopene**  **Stereoisomers** | **Retention Time (min)** | | | **Absorbance (nm)** | | |
|  | **Lycopene Stnd** | ***tangerine* (*t*^3183^)** | | **Lycopene Stnd** | ***tangerine* (*t*^3183^)** | |
| di-*cis*-lycopene 1 | 15.519 (1) | | n.d. | 432, 462, 492 |  | |
| di-*cis*-lycopene 2 | n.d. | | n.d. |  |  | |
| Unknown lycopene isomer | 19.170 (2) | | n.d. | 440, 462, 492 |  | |
| di-*cis*-lycopene 3 | 19.560 (3) | | n.d. | 434, 458, 490 |  | |
| Unknown lycopene isomer | 19.744 (4) | | n.d. | 438, 463, 492 |  | |
| di-*cis*-lycopene 4 | 20.197 (5) | | n.d. | 436, 460, 486 |  | |
| 15-*cis-*lycopene | 20.575 (6) | | n.d. | 440, 466, 496 |  | |
| di-*cis-*lycopene 5 | 21.004 (7) | | 21.34 (10) | 434, 458, 488 | 435, 459, 490 | |
| Unknown lycopene isomer | n.d. | | 21.71 (11) |  | 441, 462, 491 | |
| 5,9(5’,9’)-di-*cis-*lycopene | 21.835 (8) | | 22.06 (12) | 440, 466, 496 | 436, 465, 494 | |
| 9-*cis-*lycopene | 22.057 (9) | | 22.30 (13) | 440, 466, 496 | 441, 466, 494 | |
| Unknown lycopene isomer | 22.304 (10) | | n.d. | 446, 471, 502 |  | |
| Unknown lycopene isomer | n.d. | | 23.14 (14) |  | 444, 465, 493 | |
| Unknown lycopene isomer | n.d. | | 23.63 (15) |  | 441, 467, 495 | |
| All-*trans-*lycopene | 23.590 (11) | | 23.85 (16) | 446, 472, 504 | 446, 470, 501 | |
| 5-*cis-*lycopene | 24.125 (12) | | 23.98 (17) | 446, 472, 504 | 447, 470, 499 | |
| Identification of lycopene stereoisomers in the stereomutated lycopene standard was based on Melendez *et al.* (2013). (#)’s indicate the number of the peaks in the respective chromatographs (Supplemental Figures 4E and 5). | | | | | |  |

**Figure S1.** Assessment of protein structure of tomato PDS; ZDS; and ZISO compared to CRTI from bacteria.

**Figure S2.** Representative chromatograph (285nm – best wavelength to show all peaks) of carotenoids detected in the wild type ripe tomato. Accompanied with UV spectrum for the detected carotenoids therein. 1) lutein; 2) 15-*cis-*phytoene; 3) *cis*-phytofluene; 4) 9,15,9’ tri-*cis-*z-carotene; 5) all-*trans-*b-carotene; 6) di-*cis*-lycopene 5; 7) unknown lycopene isomer; 8) 5,9(5’,9’) di-*cis*-lycopene; 9) 9(9’) *cis*-lycopene; 10) all-*trans-*lycopene.

**Figure S3.** Absence of phytoene (μg/g FW) accumulation in young leaves of *AtPDS.OE.1A, AtPDS.OE.3, AtPDS.OE.4,* and *AtPDS.OE.6* (*n*=5, Error bars +SEM)*.*

**Figure S4.** Fruit size in terms of mass (g) of *AtPDS.OE.1A, AtPDS.OE.3, AtPDS.OE.4,* and *AtPDS.OE.6* ripe fruit compared to wild type ripe fruit (*n*>10, Error bars +SEM).

**Figure S5.** Chromatographs with carotenoids detected in the *tangerine* (*t*^3183^); *AtPDS3.2;t*^3183^; and *hp1*;*t*^3183^ ripe tomatoes. A) 285nm; B) 348nm C) 398nm; D) 437nm; E) 464nm; F) 450nm. Accompanied with UV spectrum for the detected carotenoids therein. 1) 15’*cis-*phytoene 2) *cis*-phytofluene; 3) unknown ζ-carotene isomer 1; 4) 9,15,9’ tri-*cis-*ζ-carotene; 5) unknown ζ-carotene isomer 2; 6) 9,9’ di-*cis-*ζ-carotene; 7) prolycopene; 8) Neurosporene 1; 9) Neurosporene 2; 10) di-*cis-*lycopene 5; 11) unknown lycopene isomer; 12) 5,9(5’9’) di-*cis-*lycopene; 13) 9(9’) *cis-*lycopene; 14) unknown lycopene isomer; 15) unknown lycopene isomer 16) all-*trans-*lycopene; 17) 5(5’) *cis-*lycopene; 18) lutein.

**Figure S6.** Tissue type negatively affects the extent of *AtPDS* over-expression in *tangerine* mutant flower.

(a) *Cis-*carotenoid content (μg/g FW) from the poly-*cis*-transformation of phytoene to all*-trans-*lycopene in *t*^3183^ and *AtPDS3.2*; *t*^3183^ anthesis flowers (*n*=5, Error bars +SEM).

(b) Total xanthophyll and carotenoid content (μg/g FW) in *t*^3183^ and *AtPDS3.2*; *t*^3183^ anthesis flowers (*n*=5, Error bars +SEM).

(c) Visual phenotypes of anthesis flowers.

**Figure S7.** Representative chromatograph (454nm) of stereoisomers of lycopene red, all-*trans-*lycopene standard (4oC) and blue, stereomutated all-*trans-*lycopene (80^o^C for 1hr). Accompanied with UV spectrum for the detected carotenoids therein. 1) di-*cis-*lycopene 1; *2*) unknown lycopene isomer; 3) di-*cis-*lycopene 3; 4) unknown lycopene isomer; 5) di-*cis-*lycopene 4; 6) 15 *cis-*lycopene; 7) di-*cis*-lycopene 5; 8) 5,9(5’,9’) di-*cis*-lycopene; 9) 9(9’) *cis-*lycopene; 10) unknown lycopene isomer; 11) all-*trans-*lycopene; 12) 5(5’) *cis-*lycopene.

Figure S1

Figure S2

Figure S3

Figure S4

Figure S5

Figure S6

Figure S7
